# Research and Remedies

**Published:** 1914-04-04

**Authors:** 


### RESEARCH AND REMEDIES.

### Gestational Vomiting.

Dr. Rufus Kingman contributes to *American Medicine* a vehement article in support of Graily Hewitt's method of treating the vomiting of pregnancy. He says that he has hardly ever known it fail, and uses a good deal of strong language concerning those who will not listen to his advice. Briefly, he scouts both the neurotic and toxæmic theories of this condition, and maintains that reflex disturbance is its essential origin. In treatment he advises that the patient should, after loosening all clothes, including corsets, assume the knee-elbow position for a short time; at the same time the perinæum is to be retracted digitally or with a speculum, so that air may obtain access to the vagina and the uterus tend to fall from the pelvis towards the abdomen. This process is especially recommended last thing at night, after which the patient is to extend herself face downwards on the bed and turn gradually into a comfortable posture for sleep. If this system does not at once succeed the principle is pushed further by the use of wool tampons soaked in antiseptic, which are packed gently into the vagina in the knee-elbow posture and left there for twenty-four or thirty-six hours. Dr. Kingman asserts that for the last twenty years he has never failed to cure the vomiting of pregnancy in one week by this simple method.

### Appendicitis Simulating Meningitis.

In the *British Journal of Children's Diseases* Mr. R. H. A. Whitelocke narrates a curious case in which the perforation of the appendix by a colony of thread-worms and their adventures on the peritoneum, produced symptoms in a child strongly suggestive of tuberculous meningitis. The patient was five and a half years old, and on admission to hospital had a temperature of 102° F. and a pulse rate of 112. There were marked strabismus, contraction of the pupils, and photophobia. She had vomited, screamed, and been constipated for thirty-six hours; refused food, and had incontinence of urine. Two days later flexion of the right leg and tenderness in the right iliac fossa were first noticed, with increasing sickness and accelerating pulse. Operation revealed a perforated appendix with a thread-worm actually wriggling through the opening. Dead and living parasites strewed the neighbouring peritoneum, and the appendix itself was full of them. Following this operation all symptoms subsided rapidly, and convalescence was speedy. The original diagnosis of tuberculous meningitis seemed to be rendered probable by the mimicry of the symptoms which was quite tolerably exact.

### Benzol for Leukæmia.

Some three years ago three girls who had been severely poisoned by benzol were treated at the Johns Hopkins Clinic, Baltimore. They exhibited a grave anæmia, with hæmorrhages, and two of them died. Dr. Selling, who studied the tissues of these two patients, noticed a considerable aplasia (deficiency) of their bone-marrow, and published his observation. Professor Korányi, of Budapest, was struck by this fact, and thought of the possibility of doing good to cases of myelogenous leukæmia, in which there is great over-development of the bone-marrow, by administering benzol. He was soon able to publish some remarkably good results, which have been confirmed in Chicago and in Europe. At Baltimore, naturally, considerable interest has been taken in Korányi's work, and Drs. Barker and Gibbes now report an equally striking case. A man of fifty-seven years had been suffering from this serious disease for about two months. He was treated at first with increasing doses of Fowler's solution and with salvarsan; in two and a half weeks his white corpuscles diminished from 250,000 to 210,000 per c.m. He was then put on benzol treatment, and in two months the count was 14,200, not much above normal. It seems to be established that for the first three weeks of the benzol treatment not much change takes place; but after that improvement is rapid. In this case it is by no means certain that cure has resulted, and the salvarsan might possibly have something to do with the improvement which has occurred. It is clear, however, that this new treatment for a disease hitherto incurable deserves a fair trial.

